# The Medical Schools of the United Kingdom

**Published:** 1915-09-04

**Authors:** 


### THE MEDICAL STUDENT AND HIS WORK.

### THE MEDICAL SCHOOLS OF THE UNITED KINGDOM.

**University of London, University College** (Faculty of Medical Sciences) : University Centre for Preliminary and Intermediate Medical Studies.—The College Faculty of Medical Sciences comprises the departments of physics, chemistry, botany, and zoology (the preliminary medical sciences), also the departments of anatomy, physiology, and pharmacology (the intermediate medical sciences), and the departments of hygiene and public health, and pathological chemistry (post-graduate study). Composition Fees: For the courses required by the University of London: (1) For the first medical course, 26 guineas, entitling to one attendance. (2) For the second medical course, 58 guineas if paid in one sum; 60 guineas if paid in two instalments of 30 guineas each. This fee entitles to attendance on anatomy and physiology during three years, and to one attendance on organic and applied chemistry, pharmacology and materia medica, and to the privileges of the Union Society (including the use of the gymnasium and the athletic ground at Perivale) for two sessions. For the medical education required by the Examination Board in England and the Society of Apothecaries: First examination, Parts I., II., III., 21 guineas, entitling to one attendance and to the privileges of the Union Society for one session. First examination, Part IV., and second examination, 58 guineas, if paid in one sum, and 60 guineas if paid in two instalments of 30 guineas each. This fee entitles to attendance during three years (including one attendance on each practical course in physiology) in all subjects but practical pharmacy, the fee for which is three guineas, and to the privileges of the Union Society (including the use of the gymnasium and the athletic ground at Perivale) for two sessions.

The new chemical laboratories, which occupy a large area in Gower Place on the north side of the College, will be occupied in October. The accommodation for the teaching of chemistry to medical students in their first year will be in the new laboratories.

**Charing Cross Hospital.**—This hospital is fully organised for University teaching, and contains 300 beds. The courses of study are specially designed to meet the requirements of the University of London degrees, the Conjoint Board diplomas, and the final studies of the Universities of Oxford and Cambridge. Fully equipped laboratories are available for pathological, bacteriological, and public-health work. Fees: For general students—Sessional payments: Entrance fee 10 guineas and 25 guineas annually for University students, 22 guineas annually for Conjoint Board students, so long as the student remains in the school. Special arrangements are made to permit of fees being paid in advance. The usual resident and students' appointments are open to students, and several valuable prizes are awarded annually by the school. Full information respecting the Medical School, and the course of study recommended, may be obtained on application to Dr. W. J. Fenton, F.R.C.P., Medical College, Charing Cross Hospital, London, W.C.

**Guy's Hospital.**—This hospital contains 644 beds, and has a large Medical School. There are numerous special departments, and a large number of student and resident appointments available. Special classes are held for the London University, Oxford, Cambridge, and Fellowship courses. Scholarships and prizes to the amount of £800 are awarded annually. For further particulars application should be made to the Dean of the Medical School, Mr. L. Bromley.

**King's College Hospital Medical School (University of London).**—The new King's College Hospital is situated at Denmark Hill, London, S.E., and at present contains 350 beds. The number will be increased eventually to 600. Although the hospital was opened so recently as October 1913, there are about 3,500 attendances per week in the casualty and out-patient departments. The hospital possesses every modern requirement for both patients and students. The new buildings of the Medical School, designed by Mr. W. A. Pite, F.R.I.B.A., and containing fine class-rooms, library, lecture theatre, pathological laboratory, common room, etc., will be open for the coming winter session, which will begin on October 1. In spite of the absence of a few members of the teaching staff on active service in France and at the Dardanelles, arrangements have been made to carry on the curriculum of the school with the same high standard of efficiency as prevailed before the outbreak of the war. The teaching of the preliminary subjects, including chemistry, physics, biology, anatomy, and physiology, is given at King's College, in the Strand, students coming to the hospital for their advanced work. Sixteen resident appointments are made every year. The composition fee for advanced medical studies is 70 guineas if paid in one instalment, and 72 guineas if paid in two instalments. The composition fee for the whole University or Conjoint course is 150 guineas. University Entrance Scholarships are competed for this month (September). Full particulars of these, and the new prospectus, may be obtained by application to either of the following: H. Willoughby Lyle, M.D., B.S. (Lond.), F.R.C.S., *Dean*; S. C. Ranner, M.A. (Cantab.), Secretary of the Medical School, King's College Hospital, Denmark Hill, London, S.E.

**London Hospital Medical College.**—The London Hospital is the largest institution of its kind in England, and contains 922 beds. Being situated in the East End, it ministers to a very large and poor district. The total number of patients during 1914 was 188,801, made up of 18,310 in-patients and 170,491 out-patients. During the year 6,484 major operations were performed in the theatre, while the number of operations under anæsthesia was 22,938. Five entrance scholarships are offered annually, including the Price Scholarship in science, of £100; the Epsom Scholarship; and the entrance scholarships in science, anatomy and physiology, and arts. The annual inclusive fee is 30 guineas. Appointments: Registrars, resident accoucheurs, house physicians, house surgeons, etc. One hundred and forty-one appointments are made annually. All appointments are free to full students, and all resident appointments have board free. Full information may be obtained from the Dean of the Medical College. A Hostel for students is provided on the hospital ground. The Clubs Union Athletic Ground is within easy reach of the hospital.

**London Hospital Dental School.** — The school, which is fully equipped on the most modern lines and with the latest appliances, is an integral part of the college and hospital, and is admirably adapted for the purpose of teaching. The school possesses, in addition to the theatres, laboratories, and museums of the college, a special museum of dental anatomy and surgery, operative dentistry, prosthetic and extraction rooms, and laboratories for practical dental metallurgy, dental prosthesis, and porcelain work. A systematic course of instruction in dental prosthesis is arranged for pupils. The up-to-date laboratory contains every modern apparatus, and is in charge of a skilled curator and his assistants. Students in the school will enjoy the great advantage of pursuing the medical portion of the dental curriculum in a well-equipped and successful college and hospital, of which the Dental School is part. Lecturers: Chemical and Dental Metallurgy, Hugh Candy; Physics, A. H. Fison; Human Anatomy, Professor W. Wright; Physiology, Professor E. P. Cathcart; Dental Surgery and Pathology, F. M. Farmer; Operating Dental Surgery, G. Northcroft; Operating Dental Prosthesis, G. P. Pollitt; Odonto-prosopic Orthopædics, H. Chapman; Dental Anatomy, Professor W. Wright; Dental Microscopy, E. Sprawson; Dental Bacteriology, Professor W. Bulloch; Dental Materia Medica, O. Grünbaum; Anæsthetics, R. J. Probyn-Williams.

**London (Royal Free Hospital) School of Medicine for Women.**—The Royal Free Hospital is in Gray's Inn Road, and the London School of Medicine for Women is in Hunter Street, Brunswick Square, W.C. There are 184 beds in the hospital, all of which are available for clinical teaching. A new wing has been opened containing new and enlarged departments for out-patients, casualty, midwifery (eleven beds), *x*-ray and electrical work, massage, and dentistry. Also new and enlarged students' quarters. The extension of the school is also in progress. There are numerous scholarships and prizes offered annually in connection with the school, among which may be mentioned the Isabel Thorne Scholarship of £30, the St. Dunstan's Medical Exhibition of £60 a year for three years, extendible to five years, the Mabel Sharman-Crawford Scholarship of £20 a year for four years, and the Agnes Guthrie Bursary for dental students of £60. Various resident appointments at the Royal Free Hospital are available for students of the school after qualification. Five hospitals in London are entirely officered by medical women. The session will begin on Friday, October 1. Dr. Florence Willey will deliver the inaugural address on that day at 4 P.M.

**Middlesex Hospital.**—The hospital and medical school are situated in Mortimer Street, and only a few minutes' walk from Goodge Street Station (Hampstead and Charing Cross Tube), Oxford Circus Stations (Bakerloo and Central London Tubes), and Portland Road Station (Metropolitan Railway). The hospital contains 440 beds, including special wards for cancer cases, maternity and gynæcological cases, and for diseases of the skin and eye. The cancer wing (containing ninety beds) and special investigation laboratories offer unrivalled opportunities for the study of cancer, both in its clinical and pathological aspects. In the electro-therapeutical department students obtain instruction in the treatment of lupus and cancer by the *x*-ray method. An electro-cardiographic department has recently been established. The Bland-Sutton Institute of Pathology contains a new lecture theatre, large laboratories for pathological, bacteriological, and clinical investigation, and smaller rooms for original research work. The Anatomical and Pathological Museum has been rebuilt and now forms part of the institute. There is a residential college for a limited number of students. Six house physicians, eight house surgeons, two obstetric and gynæcological surgeons, two casualty medical officers, two casualty surgical officers, and two resident officers to the special departments are appointed annually, without extra fee of any kind. Non-resident qualified clinical assistants are appointed to assist in the various out-patient departments. Three entrance scholarships, value £100, £50, and £25, and a University scholarship, value £50 (for Oxford and Cambridge students), are awarded annually in September. The successful candidates are required to become general students of the school. Numerous scholarships, medals, and prizes, to the value of over £1,000, are open for competition among students of the school. All students join the Amalgamated Students' Club, which includes the following: The Medical Society, the Common Room Society, the Cricket Club, the Football Clubs, the Athletic Clubs, the Rowing Club, the Musical Society, the Chess Club, the Lawn Tennis Club, and the Hockey Club. The athletic ground, which is eight acres in extent, is situated within easy access of the hospital—at Park Royal. There is a gymnasium within the precincts of the hospital. The winter session 1915-16 will open on Friday, October 1, at 3 P.M. The introductory address will be delivered by John Cameron, Esq., M.D., D.Sc., F.R.S.E., M.R.C.P., after which the prizes will be distributed. The annual dinner will not be held. For prospectus and full particulars application should be made to the Dean of the Medical School.

**St. Bartholomew's Hospital Medical School.**—St. Bartholomew's is the oldest and second largest hospital in London. The medical school is complete in every department, and provides lectures and practical laboratory teaching in the preliminary sciences and intermediate subjects, as well as in the purely professional and clinical parts of the curriculum. Scholarships and prizes to the total value of £1,000 are awarded annually.

**St. George's Hospital.**—The winter session commences on October 1. Students can enter at any time. Special courses are given, in which the requirements of University and other examinations receive careful attention. The hospital contains 350 beds, and has a convalescent hospital of 100 beds at Wimbledon. The usual resident and students' appointments are open to students. Scholarships and endowed prizes, amounting to £463, are awarded every year. The entire teaching and laboratories are now devoted to purely clinical subjects, as in other Universities, to the great advantage of students in their fourth and fifth years of study. Arrangements have been made with the University of London for students who enter St. George's during the first, second, or third year of the curriculum to carry out the necessary courses of instruction at either University College or King's College. Students have therefore the advantages of the lectures and practical classes of these Colleges of the University during the preliminary and intermediate portions of their studies, and then complete their course in a school entirely devoted to medicine, surgery, and the study of disease. The St. George's Hospital Club has its athletic ground of 13½ acres on the Atkinson-Morley Hospital estate at Wimbledon. It provides a football and hockey ground and six tennis courts. Further particulars may be obtained on application to the Dean of the Medical School, Dr. R. Salusbury Trevor.

**St. Mary's Hospital Medical School.**—St. Mary's Hospital contains at present 305 beds. Systematic clinical teaching is given daily in the wards and out-patients' department and in the various special departments of the hospital. The Medical School provides for the entire curriculum, and contains well-organised departments for preliminary scientific subjects. The composition fee for the whole curriculum is £140, or 60 guineas for the clinical curriculum only.

**University College Hospital.**—The Medical School comprises the departments of medicine and clinical medicine, surgery and clinical surgery, midwifery and gynæcology, pathology and morbid anatomy and clinical pathology, cardiography, bacteriology, mental physiology and mental disesases, dental surgery, practical pharmacy, and other departments for the study of special diseases, such as those of the eye, skin, ear, and throat, and for instruction in the use of anæsthetics, and in electro-therapeutics and the application of the *x*-rays. Scholarships, exhibitions, and prizes to the value of over £1,000 are offered for competition every year. Composition fees for the courses required by the University of London: For the final M.B., B.S. course, 80 guineas, or in two instalments of 50 and 32 guineas; for the medical education required by the Examining Board in England and the Society of Apothecaries, for the course required for the third examination, 80 guineas, or in two instalments of 50 and 32 guineas. The National Dental Hospital and School has recently been taken over as the Dental Department of University College Hospital. All students joining the Dental Department will do so as students of University College Hospital. The fee for the complete dental curriculum is 180 guineas, or by instalments of 62, 41, 41, 41 guineas. Qualified medical men wishing to obtain the special dental diploma of the Royal College of Surgeons can be admitted for the necessary two years' curriculum, including practical dental mechanics, operative work, and all the required dental lectures, for a fee of £120.

**Westminster Hospital Medical School.**—Westminster Hospital was the first institution of its kind in London which was founded by the voluntary contributions of the public. There is accommodation for upwards of 200 in-patients. There is a Students' Clubs Union, the subscription for membership of which is included in the general fees. The annual composition fee is 25 guineas. Special terms are given to sons of medical men. Five Entrance Scholarships are offered for competition amongst students entering in October and two for those entering in May. Sessions begin: October 1, January 6, and May 1. Four members of the Medical Staff are serving with H.M. Forces abroad; their duties as teachers in the Medical School will be undertaken by their colleagues who remain.

### PROVINCES.

**Birmingham University (Faculty of Medicine).**—In order to obtain the degrees of M.B. and Ch.B. five examinations have to be passed. The first is in chemistry, physics, and elementary biology; the second in anatomy and physiology; the third in general pathology, bacteriology, and materia medica and pharmacy; the fourth in forensic medicine, toxicology, public health, therapeutics, and special pathology; and the fifth, or final, in medicine, surgery, midwifery, diseases of women, mental diseases, and ophthalmology. The University also grants degrees and a diploma in dental surgery, and a diploma (D.P.H.) and a degree in Public Health (B.Sc.). The General Hospital and the Queen's Hospital, containing between them over 500 beds, are amalgamated for the purpose of clinical instruction, which is carried on under the direction of the University Clinical Board. Medical students also have free access to clinical instruction at the Eye, Ear and Throat, Orthopædic and Spinal, Women's, and Children's Hospitals, which are associated with the University. The composition fee for the whole course of lectures and instruction at the University is £85, besides which there is a composition fee for attendance on the medical and surgical practice and the clinical lectures at both the General and Queen's Hospitals of £42, making a total of £127. Dean: Professor Peter Thompson, M.D. The winter session opens on Tuesday, October 5, 1915.

**Bristol University.**—The University grants the conjoined degrees of M.B., Ch.B., the degree of Doctor of Medicine (M.D.), Master of Surgery (Ch.M.), Bachelor of Dental Surgery (B.D.S.), and Master of Dental Surgery (M.D.S.); also diplomas in Public Health (D.P.H.), in Dental Surgery (L.D.S.), and in Veterinary State Medicine (D.V.S.M.). For the conjoined degrees of M.B., Ch.B., the curriculum occupies five years and a half. The University lectures and laboratory courses are attended in the large and well-equipped new wing of the University. Hospital practice and clinical instruction are provided in the allied hospitals, which together afford upwards of six hundred beds and a very large external midwifery department. Three years at least must be spent in the University, and two of them subsequently to passing the second examination. Women are admitted to all degrees and diplomas and to the courses of study necessary for them. The winter session opens on October 1, 1915.

**Cardiff University College.**—Since it was founded in 1883, University College, Cardiff, has prepared students for the First Medical examination of the University of London, and for the corresponding examinations of other licensing bodies. Chairs of Anatomy, Physiology, and Pathology and Bacteriology, and Lectureships in Materia Medica and Pharmacy and Histology and Embryology have been established, making it possible to spend at least three out of the five years of the medical curriculum at this school. Arrangements have been made with the managing committee of King Edward VII.'s Hospital, Cardiff, to give students of the college the privilege of attending this hospital, which contains 196 beds. The composition fee for the three years' course of study for the Preliminary Scientific and Intermediate Examination for the London M.B. is £65.

**Durham University.**—For students who intend to graduate at Durham University it is necessary that one of the five years of professional education should be spent in attendance at the College of Medicine, Newcastle-upon-Tyne. During the year so spent the student must attend lectures at the College and the medical and surgical practice and clinical lectures at the Royal Victoria Infirmary, which contains over 400 beds. No residence at Durham is necessary, the Medical Faculty being in Newcastle-upon-Tyne. The remaining four years of the curriculum may be spent at Newcastle-upon-Tyne or at one or more of the recognised medical schools. The composition fee for the complete course of lectures is 80 guineas, and for the medical and surgical practice of the hospital 35 guineas. There are four professional examinations for the M.B., B.S. degrees. Fees, £30. Address, Dr. Robert Howden, The College of Medicine, Newcastle-upon-Tyne.

**Leeds University (School of Medicine).**—This University grants the degrees of M.B. and Ch.B., M.D. and Ch.M., M.Ch.D. and B.Ch.D., and in association with the Universities of Liverpool, Manchester, and Sheffield holds a Matriculation examination. Clinical instruction is given in the General Infirmary, which contains 520 beds, and is amply provided with special departments. Students of the University also have access for instruction to the Leeds Public Dispensary, the City Fever Hospitals, the Hospital for Women and Children, the Leeds Maternity Hospital, and the West Riding Asylum at Wakefield. Diplomas in Public Health, Psychological Medicine, and Dental Surgery are also granted.

**Liverpool University (Faculty of Medicine).**—The University grants degrees in Medicine (M.B.; M.D.), in Surgery (Ch.B.; Ch.M.), and in Hygiene (M.H.). Candidates for degrees are required to pass the Matriculation examination or an equivalent. Students may study in the University for the degrees or for the examinations of other licensing bodies. The laboratories are modern and very well equipped, and the facilities for instruction are most complete. Courses of instruction in tropical diseases can be obtained at the Liverpool School of Tropical Medicine. Fellowships, scholarships, and prizes of over £1,000 are awarded annually, in addition to entrance scholarships. The Clinical School of the University now consists of four general hospitals—the Royal Infirmary, the David Lewis Northern Hospital, the Royal Southern Hospital, and the Stanley Hospital; and of five special hospitals—the Eye and Ear Infirmary, the Hospital for Women, the Infirmary for Children, St. Paul's Eye Hospital, and St. George's Hospital for Skin Diseases. These hospitals contain in all a total of 1,127 beds. The organisation of these hospitals to form one teaching institution provides the medical student and the medical practitioner with a field for clinical education and study which is unrivalled in extent in the United Kingdom. The University also grants a diploma and degrees in dental surgery, a diploma in public health, and a diploma in tropical medicine. The Dental Hospital was opened in January 1910, and its equipment for teaching purposes is complete.

**Manchester, Victoria University (Faculty of Medicine).**—The medical department of the University is particularly well provided with facilities for instruction in all the courses for degrees and the professional qualification and for teaching the preliminary subjects—namely, anatomy, physiology, pathology, materia medica, biology, physics, and chemistry. Clinical instruction is provided at the Manchester Royal Infirmary, which contains 592 beds, and also has large out-patient and casualty departments. Associated with the Infirmary are a Convalescent Hospital at Cheadle, containing 136 beds, and the Royal Lunatic Hospital, accommodating 430 patients. Clinical instruction in gynæcology is also given at St. Mary's Hospital, and other hospitals.

**Sheffield University (Faculty of Medicine).**—The degrees of the University, M.B., Ch.B., M.D., Ch.M., and the diploma in public health are open to men and women alike. A candidate for the degrees of M.B., Ch.B. must produce certificates that he will have attained the age of 21 years on the day of graduation; that he has pursued the courses of study required by the University Regulations during a period of not less than five years subsequently to the date of his matriculation or exemption from matriculation, three of such years at least having been passed in the University, one at least being subsequent to the passing of the first examination. The University is within easy reach of the various hospitals with which it is connected for clinical purposes. The composition fee for University lectures and practical classes is £80, payable in three instalments. Composition fee for medical and surgical hospital practice (except pharmacy) for the full period required by the University and the various Examining Boards, £49 17s. 6d., payable in three instalments. The University of Sheffield offers many valuable scholarships and prizes to students in the faculty of medicine. During the coming year certain modifications in the hours of attendance for lectures, etc., will be necessary on account of the war, but the curriculum will be carried out in full, as it has been during the present year.

### SCOTLAND.

**Carnegie Trust and the Payment of Class Fees.**—This is a fund to provide under certain regulations eligible students with all or a portion of the class fees of the curriculum. Applicants must be over sixteen years of age, of Scottish birth or extraction, and must have obtained the Leaving Certificate of the Scotch Education Department or recognised equivalent. Forms of application are to be obtained from the Secretary, Carnegie Trust for the Universities of Scotland, 22 Hanover Street, Edinburgh.

### EDINBURGH.

**University of Edinburgh.**—Four degrees in medicine and surgery are conferred by the University, namely, Bachelor of Medicine (M.B.), Bachelor of Surgery (Ch.B.), Doctor of Medicine (M.D.), and Master of Surgery (Ch.M.). The degree of Ch.B. is not conferred on any person who does not at the same time obtain the degree of M.B., and the degree of M.B. is not conferred on any person who does not at the same time obtain the degree of Ch.B. A University diploma is granted in Tropical Medicine and Hygiene (D.T.M. and H.), and a University certificate is granted in Diseases of Tropical Climates. There has also been recently formulated regulations for a Diploma in Psychiatry.

To obtain the Edinburgh medical degree it is necessary to pass the preliminary examination or its recognised equivalent, and to pass four professional examinations. The first is in botany, zoology, physics, and chemistry; the second in anatomy and physiology; the third in pathology, materia medica, and therapeutics; and the final in surgery, clinical surgery, medicine, clinical medicine, midwifery, clinical gynæcology, forensic medicine, and public health. In order to obtain the M.B., Ch.B. degrees, it is necessary to spend at least two of the five years of medical study in the University of Edinburgh. Clinical instruction is given in the Royal Infirmary, which contains nearly 900 beds; Royal Hospital for Sick Children, with 120 beds; Maternity Hospital, with 40 beds available for clinical instruction; City Fever Hospital, with 600 beds, and the Royal Mental Hospital, Morningside, with 500 beds. The total number of beds available for the clinical instruction of students of the University is 2,160. The fees for the four professional examinations amount to £23 2s. Communications should be addressed to the Dean of the Medical Faculty, University New Buildings, Edinburgh, and letters respecting the preliminary examination to Mr. James Dowie, Clerk of Senatus, The University, Edinburgh.

**School of Medicine of the Royal Colleges**
This School of Medicine is constituted by an association of lecturers who lecture in several buildings near the Royal Infirmary. The lecturers are recognised or licensed by the Royal Colleges of Surgeons and Physicians, and also by the University. The teaching is similar to that of the Scottish Universities, and the students receive similar certificates at the close of each session. The lectures qualify for the University of Edinburgh and other Universities; the Royal Colleges of Physicians and Surgeons of Edinburgh, London, and Dublin; the Faculty of Physicians and Surgeons of Glasgow, and other Medical Boards. The whole education required for graduation at the University of London may be taken in this school. The anatomy rooms open and the lectures commence on October 5. The facilities for clinical instruction are similar to those provided for the University, and there are special classes for women. Full particulars and copy of the calendar of the school may be had gratis from the Dean of the School, 11 Bristo Place, Edinburgh.

**School of Medicine for Women.**—Precisely the same facilities for medical study are given as to male students in the School of Medicine, Edinburgh. Regulations, fees, and arrangements are the same. The lecturers are recognised by the University of Edinburgh as giving courses admitting to the degrees of M.B., Ch.B. Clinical instruction is given in the Royal Infirmary and other hospitals mentioned above. The benefit of the Carnegie Trust is open to students of the college. Most of the classes are held at Surgeons' Hall. A sitting-room is provided for the students. The office of the Dean of the School is also at Surgeons' Hall. Applications for prospectuses should be addressed to Miss Keith, Secretary, School of Medicine for Women, Surgeons' Hall, Edinburgh.

### GLASGOW.

**University of Glasgow.**—The whole course of study for the M.B., Ch.B. can be passed in the Medical School of the University. The regulations are similar to those stated above for Edinburgh. Clinical instruction is given at the Western Infirmary, which contains about 600 beds; at the Royal Infirmary, which contains over 600 beds; and also at the Lunatic Asylum, the Eye Infirmary, the Maternity Hospital, the City Fever Hospitals, and various other hospitals. The cost of the course, including matriculation, fees for classes, for hospital attendance, and for professional examinations, amounts to about £150. The normal arrangements at Glasgow University for the instruction of medical students are not likely to be very greatly affected by the war. The total number of students will be somewhat diminished owing to students joining the fighting units, but students of the fourth and fifth years will in nearly all cases continue their studies without interruption in order to obtain their qualifications as soon as practicable in accordance with the advice of the Army Medical Service. Some of the members of the teaching staff are engaged in the war, but their places in the work of the medical school will be taken by others.

**Anderson College of Medicine.**—The college, at which a full medical curriculum can be obtained, is situated in Dumbarton Road, adjoining the Western Infirmary and the University. Degrees and diplomas: Certificates of attendance on the lectures are accepted by the Universities of London and Durham, by the Universities of Glasgow, Edinburgh, etc., under conditions stated in the calendars, and by all the Royal Colleges and licensing boards in the United Kingdom. The public-health course qualifies for the Scottish Licensing Board, the Irish Colleges, and the Universities of Oxford, Cambridge, London, etc. The Carnegie Trust extends its benefactions to students of Anderson's College. Particulars may be obtained from Sir W. S. McCormick, LL.D., the Carnegie Trust Offices, Edinburgh. A calendar will be sent on receipt of a postcard by the Secretary to the Medical Faculty, The Anderson College of Medicine, Glasgow, W.

**Queen Margaret College (the Women's Department of the University).**—This is an integral part of the University of Glasgow. The courses, regulations, and fees are the same as for men. The instruction is given by University professors and lecturers appointed by the University Court, partly in mixed and partly in separate classes. The College provides a separate building, with class-rooms, laboratories, library, and other teaching appliances. The women have all the rights and privileges of University students. Clinical instruction is provided in the Royal Infirmary and in other hospitals.

**St. Mungo's College: the Medical School of the Glasgow Royal Infirmary.**—The College buildings are situated within the grounds of the infirmary, and are provided with class-room and laboratory accommodation for several hundred students. Students receive their clinical instruction in the wards of the infirmary, which contains 600 beds, and in the Glasgow Maternity and Women's Hospital. The College provides a complete medical curriculum, and the classse qualify for the diplomas of the English, Scottish, and Irish Conjoint Boards, and under certain conditions for the various Universities. Students who have fulfilled the conditions of the Carnegie Trust are eligible for the benefits of this Trust during the whole course of their studies at St. Mungo's College. The classes at St. Mungo's College are open to male and female students equally. Students entering for the Dental diploma can take out almost all their classes at St. Mungo's College. There is a special department of public health, attendance on which qualifies for the Conjoint Boards and the Universities of Oxford and Cambridge. The inclusive fees for the whole Medical curriculum amount to about £70.

**Western Medical School.**—This School is situated in University Avenue, opposite to the principal gate of the University. Lectures and demonstrations are given in the usual winter and summer sessions on anatomy, surgery, medicine, midwifery and gynæcology, ophthalmology, dermatology, and diseases of throat and nose. Some of the classes qualify for graduation and for Scottish diplomas. The usual class fee is £2 2s. There is no Matriculation fee. Further information may be obtained from the Secretary.

### ABERDEEN.

**University of Aberdeen.**—The winter session commences on Thursday, October 14, when it is expected that classes will meet in all the ordinary subjects qualifying for graduation in Medicine and Surgery. The regulations are practically the same as those of the other Scottish Universities. Clinical instruction is obtained at the Royal Infirmary, which contains 270 beds, in the Sick Children's Hospital, and several other institutions, including the Royal Lunatic Asylum and the City Fever Hospital. At least two of the five years of study and at least six of twelve specified subjects must be spent or taken in the University. The remaining three years may be spent and the remaining subjects taken in any University of the United Kingdom, or in any Indian, Colonial, or foreign University, recognised for the purpose by the University Court, or in recognised Medical Schools or under recognised teachers. There are four professional examinations, and the cost of Matriculation, class and degree fees for the whole curriculum is about £160. Address, Mr. D. R. Thom, M.A., Secretary, The University, Aberdeen.

### ST. ANDREWS.

**University of St. Andrews.**—Medical students may attend for the first two years of study either in St. Andrews or in Dundee. The remainder of the course must be taken in Dundee, and full particulars respecting the curriculum and examinations may be obtained from Professor Kynoch, Dean of the Faculty of Medicine, the Medical School, Dundee. Clinical instruction is given at the Dundee Royal Infirmary, which has 400 beds (including Maternity Hospital), at the Dundee Eye Institution, the King's Cross Fever Hospital, and the Dundee District Asylum.

### IRELAND.

**University of Dublin (Trinity College).**—The Medical School of Trinity College, or School of Physic in Ireland, was established in 1711. All classes are open to external students as well as to students of the University, provided that they have passed an examination in Arts recognised by the General Medical Council. Women students are admitted to the classes and to the University degrees. Candidates for the degrees in Medicine (M.B.), Surgery (B.Ch.), and Midwifery (B.A.O.) must be of B.A. standing, and must have spent at least five academic years, reckoned from the date of Matriculation in the study of Medicine. The Arts course may be taken concurrently with the Medical course, and the B.A. degree need not be taken before the final Medical examination, but the Medical degrees are not conferred without the Arts degree. The M.D. degree is awarded to graduates in Medicine of M.A. standing who have read an approved thesis publicly before the Regius Professor of Physic. Special classes are held for the Diploma in Public Health. The winter session begins on October 1. Further information regarding the Medical and Dental courses may be obtained by application to the Registrar, School of Physic, Trinity College, Dublin.

**Royal College of Surgeons in Ireland (the Schools of Surgery).**—These schools are carried on within the college buildings, and are specially subject to the supervision and control of the Council. The buildings have been reconstructed, the capacity of the dissecting-room nearly trebled, and special pathological, bacteriological, public health, chemical, and pharmaceutical laboratories fitted with the most approved appliances, in order that students may have the advantage of the most modern methods of instruction. There are special rooms set apart for lady students. The Medical students' guide will be forwarded post free on application to the Registrar, Royal College of Surgeons, Dublin.

**University College Medical School, Dublin.** Founded in 1855, it is now the largest medical school in Ireland. It prepares students specially for the examinations of the National University and the Conjoint Colleges of Ireland and Edinburgh, but its lectures and practical courses are recognised by all the licensing bodies in Great Britain and Ireland. In addition to the ordinary Medical examinations it prepares students for the D.P.H. and for the various higher University examinations in pathology, physiology, chemistry, etc. Eight exhibitions and numerous gold and silver medals are offered annually for competition.

### BELFAST.

**Queen's University.**—This University provides all the classes required for a complete medical curriculum. Women are eligible as students. Clinical instruction is given at the Royal Victoria Hospital, which was rebuilt a few years ago and has 300 beds, and at the Mater Infirmorum Hospital, which has 150 beds. Various other hospitals are open to the students of the University. The cost of the curriculum intended for students proceeding to the degrees of the Queen's University of Belfast is, approximately, £105. This includes examination fees and a perpetual ticket for attendance at the Royal Victoria Hospital or the Mater Infirmorum Hospital, but not fees for the special hospitals. The course for the Conjoint Board costs about the same amount. A pamphlet containing full information regarding the new regulations for courses, fees, entrance and other scholarships, etc., can be had free of cost on application to the Secretary, Queen's University, Belfast.

### GALWAY.

**University College.**—In addition to various laboratories, a dissecting-room, and anatomical lecture theatre, the College contains a large natural history and geological museum, as well as an extensive library for the students' use. New chemical and pathological laboratories are now ready for use. There are six junior scholarships of the annual value of £25 each specially reserved for students in medicine, and awarded on the results of the University examination of the corresponding year. In addition, medical students at entrance are eligible to compete at the General Entrance Scholarship examination, and to hold the scholarship, if successful, in the Faculty of Medicine. Clinical instruction is given in the Galway Hospital and in the Galway Union and Fever Hospitals. Full information as to courses of lectures, scholarships, and class fees can be obtained from the Registrar, University College, Galway.

### CORK.

**University College.**—Students can attend courses which meet the requirements of the National University of Ireland, the Conjoint Boards of London, Edinburgh, or Dublin, and other examining bodies. Clinical instruction is given at the North and South Infirmaries, each of which contains 100 beds, at the District Hospital, which contains 1,200 beds, and at other hospitals. The college is a constituent college of the National University of Ireland, and holds its own examinations in all subjects required for the degrees of that University. Thirty-six Entrance and County Council Scholarships, varying from £50 to £20, which may be held in any Faculty, are offered for competition. There are, in addition, twelve scholarships of about £30 each offered for competition to medical students of the second, third, fourth, and fifth years. The Blayney Scholarship, of about £32, is awarded to the candidate who, being of the prescribed standing, obtains highest marks with honours at the National University examination for the M.B., B.Ch., B.A.O. degrees, taken as a whole. Further information can be had on application to the Registrar, University College, Cork.

